# Neuropathic and Nociplastic Pain Profiles are Common in Adult Chronic Nonbacterial Osteitis (CNO)

**DOI:** 10.1007/s00223-024-01214-3

**Published:** 2024-04-16

**Authors:** Anne T. Leerling, Marieke Niesters, Marcel Flendrie, Marije Tel, Natasha M. Appelman-Dijkstra, Olaf M. Dekkers, Elizabeth M. Winter

**Affiliations:** 1 Division of Endocrinology, Department of Internal Medicine, Leiden University Medical Center, Leiden, The Netherlands; 2 Center for Bone Quality, Leiden University Medical Center, Albinusdreef 2, 2333 ZA Leiden, The Netherlands; 3 Department of Clinical Epidemiology, Leiden University Medical Center, Leiden, The Netherlands; 4 Department of Anesthesiology and Pain Medicine, Leiden University Medical Center, Leiden, The Netherlands; 5 Department of Rheumatology, Sint Maartenskliniek, Nijmegen, The Netherlands

**Keywords:** Chronic nonbacterial osteomyelitis, Osteitis, SAPHO, Pain, Central sensitization, Central pain, Fibromyalgia, Neuropathic pain, Nociplastic pain, Nociceptive pain, Patient reported outcomes

## Abstract

Chronic nonbacterial osteitis (CNO) is a rare musculoskeletal disease causing chronic bone pain. It is known that chronic musculoskeletal pain may involve other mechanisms than nociceptive pain only. We investigate the prevalence of neuropathic and nociplastic pain in adult CNO and their association with clinical characteristics and treatment outcomes. Survey study among the Dutch adult CNO cohort (*n* = 84/195 participated), including PAIN-detect for neuropathic pain, and the Central Sensitization Inventory (CSI), Fibromyalgia Rapid Screening Tool (FiRST), and ACTTION-APS Pain Taxonomy (AAPT) for nociplastic pain. Clinical characteristics and CNO-related bone pain scores were compared between patients with exclusive nociceptive pain and those with nociceptive pain plus neuropathic and/or nociplastic pain (mixed pain). 31% (95% CI 21–41) of patients classified as likely having neuropathic pain according to PAIN-detect. 53% (41–64) of patients displayed central sensitization on CSI, 61% (50–72) screened positive for fibromyalgia on FiRST and 14% (7–23) of patients fulfilled the AAPT criteria, all indicative of nociplastic pain. Mixed pain was associated with longer diagnostic delay (mean difference 2.8 years, 95% CI 0.4–5.2, *p* = 0.023), lower educational level (72% versus 20%, *p* < 0.001), and opioid use (37% versus 13%, *p* = 0.036). Despite comparable disease severity and extent, patients with mixed pain reported significantly higher CNO-related bone pain scores. This study demonstrates the high prevalence of mixed pain in adult CNO, in which neuropathic and nociplastic pain exist alongside nociceptive inflammatory bone pain. Disease burden in CNO may extend beyond inflammatory activity, highlighting the need for a multifaceted management approach.

**Supplementary Information:**

The online version contains supplementary material available at 10.1007/s00223-024-01214-3.

## Introduction

Chronic nonbacterial osteitis (CNO) is a rare bone disease with a heterogeneous clinical presentation, occurring in children and adults [1–3]. CNO has historically been indicated by various terms, including synovitis, acne, pustulosis, hyperostosis, osteitis (SAPHO)-syndrome and chronic recurrent multifocal osteomyelitis (CRMO). The disease spectrum is centrally characterized by relapsing–remitting sterile bone inflammation, causing bone pain, stiffness, compromised joint mobility and bone deformation [3, 4]. Despite ongoing efforts for timely diagnosis, patients with CNO still face an average diagnostic delay of 5 years during which they may be exposed to chronic pain [5, 6]. After diagnosis, chronic pain remains the main contributor to overall disease burden, reflected in frequent work absence and decreased quality of life [6–8]. Clinical management of CNO therefore focuses on alleviating pain through targeting the osteitis with various anti-inflammatory and anti-resorptive treatments, all of which are off-label due to lack of supportive evidence [1, 2, 9].

While controlling inflammation is the primary focus of treatment, studies in related musculoskeletal diseases, such as axial spondylarthritis and psoriatic arthritis, have shown that patients may continue to report pain in the absence of active inflammation [10, 11]. This implies that non-inflammatory factors also contribute significantly to overall pain and associated disease burden [12, 13]. Physiologically, (musculoskeletal) pain can be categorized into nociceptive, neuropathic or nociplastic pain [14]. Nociceptive pain arises from damage to non-neural tissue and includes pain caused by inflammation or injury. Neuropathic pain arises from peripheral or central nervous system damage and is often described as shooting, burning, or tingling and may be associated with dysesthesia or allodynia. Nociplastic pain is a relatively recently proposed pain profile thought to arise from alterations in central sensory processing and pain modulatory mechanisms. Phenotypically, nociplastic pain is more widespread and intense than would be expected from the amount of identifiable tissue damage, and is often accompanied by central nervous system symptoms such as fatigue, sleep problems, memory and mood disorders [15]. A well-known example where nociplastic pain is believed to be the main pain profile is fibromyalgia [14, 16, 17].

Both neuropathic and nociplastic pain have proven common in axial spondylarthritis, psoriatic arthritis and rheumatoid arthritis alongside nociceptive pain caused by inflammation. Their co-existence is associated with higher patient reported disease activity, poorer treatment outcomes, and lower quality of life [11, 18–20]. For adult CNO, the co-existence of these pain profiles is currently unknown, but suspected based on the notable number of patients that do not experience pain improvement despite receiving anti-inflammatory treatments [21]. This study therefore aimed to investigate the prevalence of neuropathic pain and nociplastic pain in adult CNO, to identify patients characteristics associated with these pain profiles, and to assess their influence on patient reported outcomes before and after treatment.

## Methods

### Study Design and Population

A survey study was conducted at the Leiden University Medical Center, the Dutch national expert center for CNO. The study was reviewed and approved by the institutional review board and adhered to the Consensus-Based Checklist for Reporting of Survey Studies (CROSS) guidelines [22]. Adult patients with clinical and radiologic diagnosis of CNO referred between 1993 and 2022 and with available contact information were eligible for inclusion; characteristics of this cohort have been reported previously [4]. All eligible patients were approached digitally in September 2022 to participate in the surveys (part of which pertained to physical therapy and is described elsewhere) and reminded twice as appropriate until survey closure in January 2023. Survey entry was done via a personal link preventing multiple entries from the same individual. Patients who completed the surveys provided written consent for the use of their data. Patients who declined participation or did not respond to the invitation were given the opportunity to object to the use of their electronic health record data; without objection, their data were used for a non-participant control group to assess the sample representativeness.

### Surveys

The survey set pertaining to this study included PAIN-detect to assess the probability of neuropathic pain. Its questions cover pain quality, course, and associated symptoms, aiming to identify features consistent with neuropathic pain. Responses yield a composite score that categorizes patients into “unlikely (or < 15% probability”), “uncertain” (warranting further investigation) and “likely (> 90% probability”) neuropathic pain [23]. To evaluate symptoms consistent with nociplastic pain, the central sensitization inventory (CSI) was used, which is designed to identify and quantify symptoms associated with a nociplastic pain profile, including widespread pain and central nervous system symptoms [24]. Both PAIN-detect and CSI have been validated in Dutch. Additionally, the ACTTION-APS Pain Taxonomy (AAPT) criteria for fibromyalgia and the full Fibromyalgia Rapid Screening Tool (FiRST) [25, 26] were included to specifically evaluate symptoms associated with fibromyalgia, a condition thought to be mediated by nociplastic pain mechanisms. A self-developed 16-question section regarding pain management (see S1 for full section) was also developed, and pre-tested by three patients from different sociodemographic backgrounds and evaluated for clarity, coverage of relevant topics, and survey burden, after which amendments were made according to their feedback.

### Data Collection

The following data were extracted from the medical records for participants and non-participants: demographic data, comorbidities, intoxications, work status, clinical data on diagnosis, disease course, treatment history, and pre-treatment pain scores (collected at first presentation or re-referral due to relapse). Pain scores had been collected as part of routine clinical practice, where patients were instructed to report bone pain related to CNO at the sight of known lesions, in the form of maximal, minimal, average and sleep-related pain scores over the past 7 days as obtained by Brief Pain Inventory (BPI; scores on numerical rating scale of 0–10, 10 indicating worst possible pain) [27, 28]. For participants, pain scores at 6–12 months of therapy and therapy contents were additionally collected. If multiple suitable pain score-pairs were available, the ones closest in time to survey completion (September-December 2022) were selected. Standard therapy at our center comprises non-steroidal anti-inflammatory drugs (NSAIDs), and, if ineffective, addition of intravenous bisphosphonates. All participants were classified as having nociceptive inflammatory bone pain, since this had led them to present at the CNO referral center and receive their diagnosis. Participants were further classified as having neuropathic pain when categorizing as “likely” having neuropathic pain as determined by PAIN-detect [23], and as having nociplastic pain when scoring moderate to extreme on the CSI scale, and/or a positive FiRST score, and/or meeting the AAPT criteria for fibromyalgia [24, 26, 29]. For the FiRST score specifically, a positive score was given at a modified cut-off of 3 points, as suggested by a recent study on its application and validity in adult CNO [29]. Ultimately, participants were grouped into those with a strictly nociceptive pain profile only, and those with nociceptive pain *plus* neuropathic pain and/or nociplastic pain, referred to as “mixed pain”.

### Statistical Analysis

Statistical analyses were performed using SPSS Statistics version 25 (IBM Corp., USA). Categorical data are presented as *n* and percentages, while continuous variables are represented as mean ± standard deviation (SD or range) or median (interquartile range (IQR)). Prevalence is reported as proportion with 95% confidence interval (CI). Participants and non-participant controls were compared using chi-square tests or Fisher’s exact test for categorical data, unpaired t-tests or one-way ANOVA for parametric numerical data, and Mann–Whitney *U* tests or Kruskal–Wallis tests for non-parametric data. Similar comparative analyses were done for patients with nociceptive pain only versus those with mixed pain. Pearson’s correlation coefficient was used to evaluate the correlation of CSI and PAIN-detect score with scores for inflammatory bone pain as measured by BPI. Differences in treatment outcomes between participants with a strictly nociceptive pain profile and those with mixed pain were evaluated with analysis of covariance, with pain profile as random factor, post-treatment scores as dependent variables, and pre-treatment scores as covariates. Bonferroni correction was applied to adjust for multiple testing in analyses including pain scores; level of significance is indicated per analysis as appropriate.

## Results

### Study Population

**Fig. 1:**
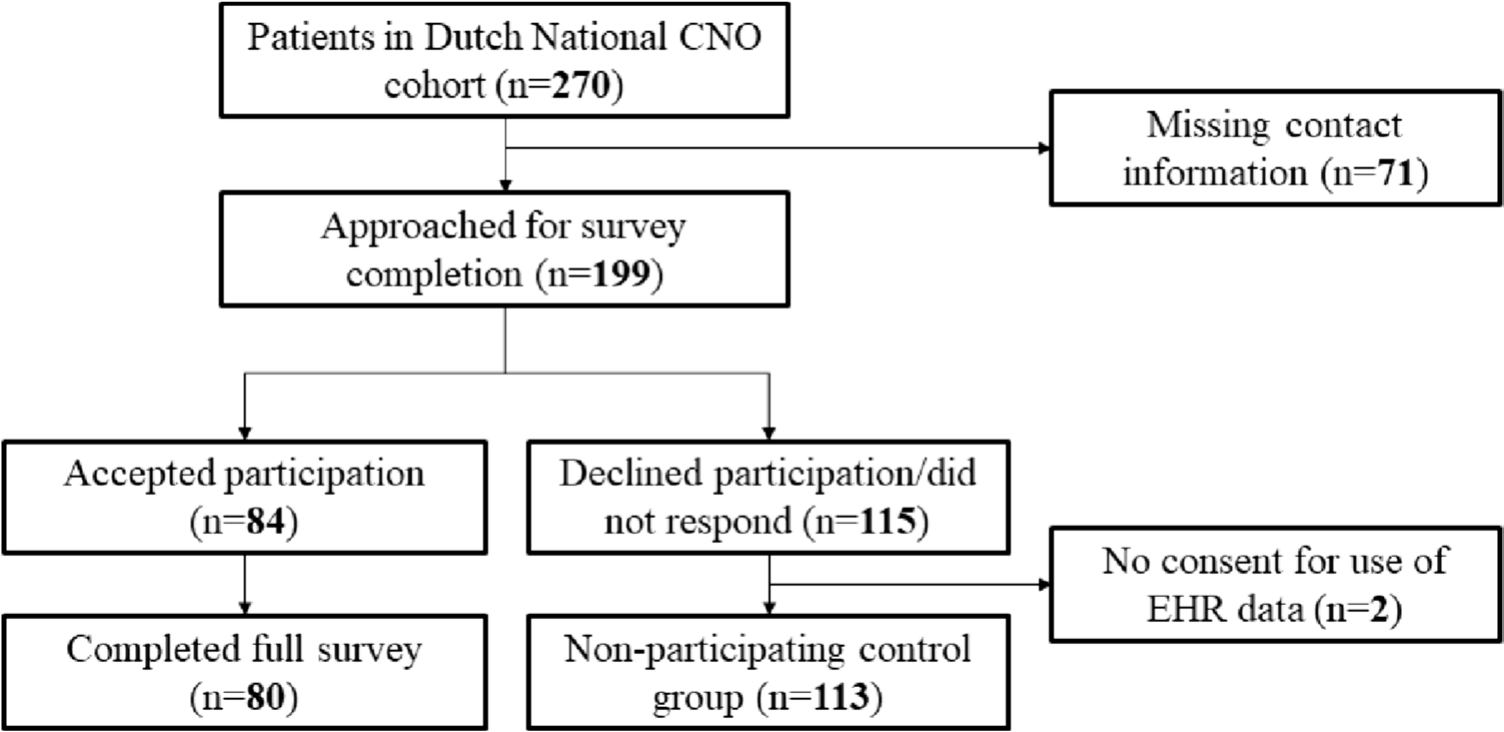
Overview of study inclusion process. *EHR* electronic health record

Of 270 patients in the Dutch adult CNO cohort, 195 had available digital contact information. Of these, 84 patients accepted participation in the survey study (response rate 43%); 80 completed the questions in full, and 4 in part (Fig. 1). Of patients who declined or did not respond, all but 2 consented to the use of their electronic health record data to form a non-participant control group. Demographics, disease status, and CNO-related bone pain scores before treatment initiation were similar across participants non-participant controls (see Table 1). Participating patients were predominately female, of middle age, and mostly suffered from bone inflammation in the anterior chest wall, all of which are known features of adult CNO.

**Table 1:** Characteristics of participants and non-participants

	Participants (*n* = 84)	Non-participants (*n* = 109)	Mean difference (95% CI)	*p*
Sociodemographic characteristics				
Gender, female, *n* (%)	79 (95%)	96 (88%)	–	0.086
Age (mean, range)	51 (27–89)	50 (38–61)	1.7 (− 2.0–5.5)	0.179
Diagnostic delay (years; mean, range)	6 (0–29)	6 (0–30)	0.5 (− 1.2–0.3)	0.274
Disease duration (years; mean, range)	14 (1–70)	15 (3–40)	0.1 (− 2.8–3.0)	0.472
Auto-inflammatory comorbidity (any), *n* (%)	23 (28%)	40 (37%)	–	0.257
Psychiatric comorbidity (any), n (%)	12 (15%)	13 (12%)	–	0.554
Diagnosis of fibromyalgia	6 (7%)	8 (7%)		0.221
Active smoking, *n* (%)	15 (20%)	28 (27%)	–	0.715
Educational level, lower, *n* (%)	28 (57%)	37 (69%)		0.232
Work absence due to CNO, *n* (%)	30 (47%)	42 (49%)	–	0.638
Disease characteristics				
Number of bones involved (mean, range)	3 (1–6)	3 (1–6)		0.739
Distribution, *n* (%)				0.348
Anterior chest wall only	56 (89%)	70 (80%)		
Anterior chest wall + spine	2 (3%)	9 (10%)		
Anterior chest wall + mandible	4 (6%)	8 (9%)		
Spine only	1 (2%)	1 (1%)		
Extra-skeletal features,* n* (%)				
Pustulosis palmoplantaris	22 (27%)	26 (24%)		0.234
Psoriasis	10 (12%)	11 (10%)		0.672
Arthritis	5 (6%)	8 (7%)		0.642
Pain treatment pre-diagnosis, *n* (%)				
NSAIDs	60 (73%)	74 (72%)		0.841
Opioids	10 (12%)	16 (16%)		0.538
Physical therapy	31 (39%)	47 (46%)		0.289
Rehabilitation	2 (3%)	1 (< 1%)		0.426
Scores for CNO-related bone pain pre-treatment (NRS 0–10)				
Maximal pain in past 7 days, mean ± SD	7.0 ± 2.0	6.9 ± 2.4	0.02 (− 0.9–1.0)	0.477
Minimal pain in past 7 days, mean ± SD	3.3 ± 2.2	4.0 ± 2.3	0.7 (− 0.3–1.8)	0.083
Average pain in past 7 days, mean ± SD	5.4 ± 2.0	5.4 ± 2.1	0.1 (− 0.9–1.0)	0.443
Sleep disturbance in past 7 days, mean ± SD	5.0 ± 3.0	5.4 ± 2.6	0.4 (− 0.9–1.7)	0.273

*NRS* numerical rating scale

### Neuropathic and Nociplastic Pain in Adult CNO

**Fig. 2:**
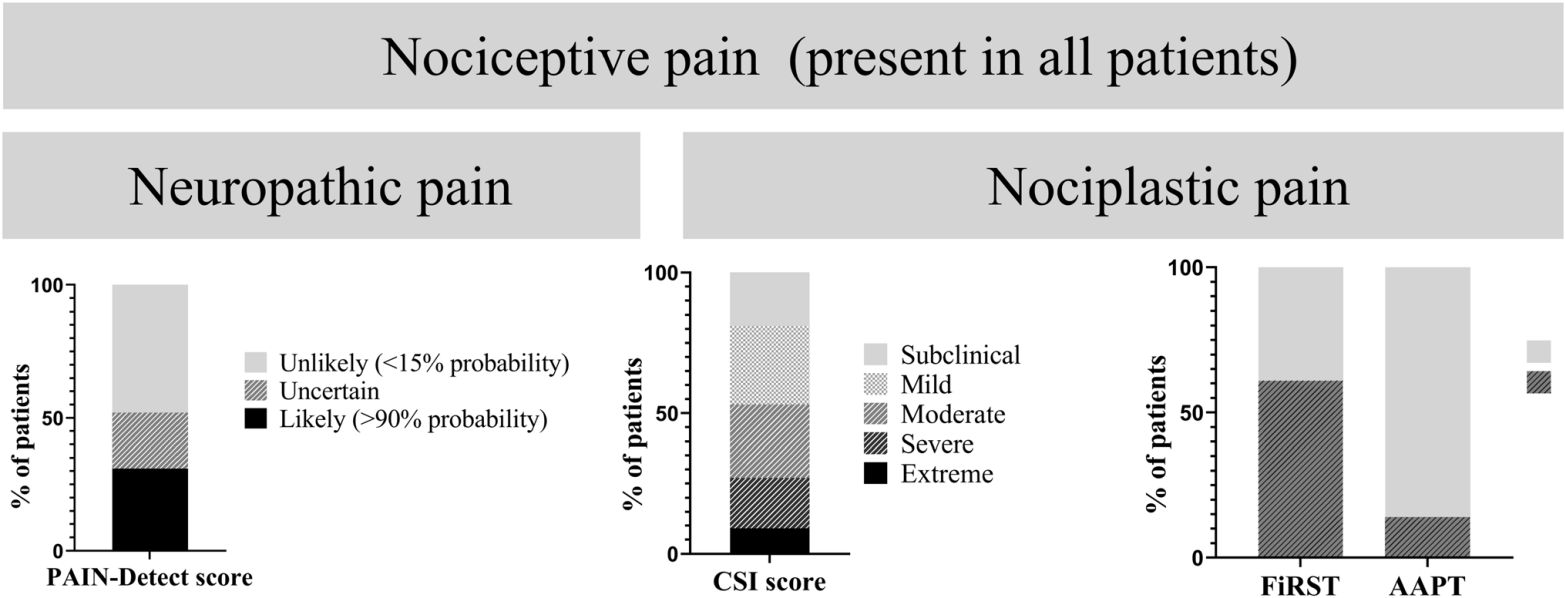
Prevalence of neuropathic pain as assessed by PAIN-detect and nociplastic pain as evaluated by CSI, FiRST screening tool and AAPT criteria for fibromyalgia in adult CNO. Nociceptive pain was assumed in all patients. *CSI* central sensitization inventory

The prevalence of neuropathic and nociplastic pain profiles in adult CNO is displayed in Fig. 2**.** PAIN-detect scores demonstrated that *n* = 25, 31% (95% CI 21–41) of patients classified as “likely (> 90% probability)” having neuropathic pain. *N* = 17, 21% (13–31) classified as “uncertain”, indicating further evaluation is necessary to make a definitive assessment. CSI revealed a mean score of 40 ± 14.3, with *n* = 42, 53% (95% CI 41–64) of patients displaying moderate to extreme degrees of sensitization, consistent with a nociplastic pain profile. Respectively, *n* = 14, 18% (10–28) and *n* = 7, 9% (4–17) of patients had severe or extreme symptoms of central sensitization. *N* = 49, 61% (50–72) of participating adult CNO patients screened positive for fibromyalgia on FiRST, whereas *n* = 11, 14% (7–23) of patients fulfilled the AAPT criteria for fibromyalgia, all of whom also had screened positive on FiRST.

Numerical scores for neuropathic pain on PAIN-detect and the presence of central sensitization on the CSI were correlated with patient reported scores for CNO-related bone pain as obtained in routine clinical practice by BPI (see S2). Central sensitization scores on CSI were positively correlated with scores for maximal bone pain, average pain, and sleep disturbance whereas neuropathic pain score on PAIN-detect was correlated with all CNO-related bone pain scores.

### Patient Characteristics Associated with Mixed Pain

**Fig. 3:**
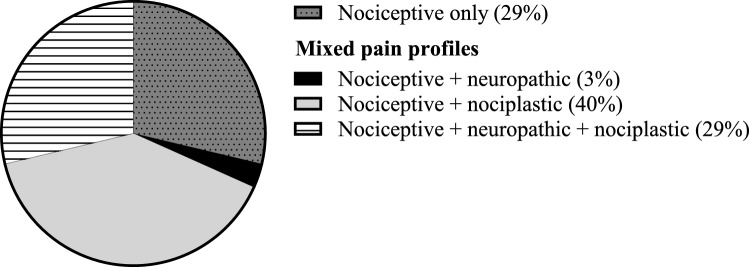
Distribution of pain profiles among adult CNO patients

Distribution of pain profiles among participating adult CNO patients is shown in Fig. 3. All patients were assumed to have nociceptive inflammatory bone pain, as this has led them to present with CNO in the first place. *N* = 23, 29% (20–40) of patients exclusively had nociceptive pain, with no indications of either neuropathic or nociplastic pain profiles. Contrarily, *n* = 2, 3% (3–9) had a combination of nociceptive and neuropathic pain, *n* = 32, 40% (30–52) had a combination of nociceptive and nociplastic pain, and *n* = 23, 29% (20–40) had nociceptive, neuropathic and nociplastic pain. Classification of nociplastic pain was most frequently based on FiRST and CSI (*n* = 27), on FiRST, CSI and fulfillment of the AAPT criteria (*n* = 10), or on FiRST and CSI only (*n* = 12 and *n* = 5, respectively). Altogether, a majority of *n* = 57, 71% (60–81) patients had additional neuropathic pain, nociplastic pain, or both, alongside nociceptive inflammatory bone pain from here on referred to as “mixed pain”.

Sociodemographic and disease characteristics of patients with nociceptive pain only and those with mixed pain (i.e., nociceptive pain and neuropathic pain and/or nociplastic pain) are depicted in Table 2. Patients with mixed pain tended to be younger than those with strictly nociceptive pain (mean difference 5.2 years, 95% CI − 11.2–0.8). The mixed pain group contained a slightly higher proportion of female patients. Conversely, 73% of female patients presented with mixed pain compared to 25% of male patients (*p* = 0.038). Mixed pain was further associated with longer diagnostic delay (mean difference 2.8 years, 95% CI 0.4–5.2, *p* = 0.023), lower educational level (72% versus 20%, *p* < 0.001), and opioid use before or after diagnosis of CNO (37% versus 13%, *p* = 0.036). Disease duration and extent, including number of CNO lesions, distribution pattern, and extra-skeletal features did not differ between groups. Scores for CNO-related bone pain, obtained before treatment initiation (baseline) were compared between patients with nociceptive pain only (*n* = 13 with available data) and mixed pain (*n* = 36 with available data). Scores for maximal pain, average pain, and sleep disturbance due to pain were numerically higher in patients with mixed pain (mean difference (95% CI) 1.9 (0.7–3.1), *p* = 0.005, 1.8 (0.2–3.3), *p* = 0.008 and 3.5 (1.9–5.0), *p* < 0.001, respectively).

**Table 2:** Sociodemographic and disease characteristics of patients with nociceptive pain only and those with mixed pain (i.e., nociceptive pain plus neuropathic pain and/or nociplastic pain)

	Nociceptive pain only (*n* = 23)	Mixed pain (*n* = 57)	Mean difference (95% CI)	*p*
Sociodemographic characteristics				
Sex, *n* (%)			–	
Female	20 (87%)	55 (98%)		
Male	3 (13%)	1 (2%)		0.038
Age (mean, range)	55 (30–79)	50 (27–68)	5.2 (− 11.2–0.8)	0.091
Diagnostic delay (years; mean, range)	4 (0–17)	7 (0–29)	2.8 (0.4–5.2)	0.023
Disease duration (years; mean, range)	15 (2–39)	13 (1–40)	2.2 (− 6.7–3.7)	0.555
Auto-inflammatory comorbidity (any), *n* (%)	7 (30%)	16 (29%)	–	0.906
Psychiatric comorbidity (any), n (%)	1 (5%)	10 (18%)	–	0.122
Diagnosis of fibromyalgia	1 (5%)	5 (9%)		0.509
Active smoking, *n* (%)	4 (17%)	11 (20%)	–	0.882
Educational level, lower, *n* (%)	3 (20%)	23 (72%)		< 0.001
Work absence due to CNO, *n* (%)	5 (29%)	23 (54%)	–	0.092
Disease characteristics				
Number of bones involved (mean, range)	3 (1–6)	3 (1–5)	0.2 (− 1.1–0.7)	0.643
Distribution, *n* (%)				0.155
Anterior chest wall only	16 (80%)	37 (93%)		
Anterior chest wall + other site	4 (20%)	3 (8%)		
Extra-skeletal features, *n* (%)				
Pustulosis palmoplantaris	7 (30%)	15 (27%)		0.777
Psoriasis	3 (13%)	7 (12%)		0.926
Arthritis	0 (0%)	5 (6%)		0.280
Opioid use (current or past), *n* (%)	3 (13%)	21 (37%)		0.036
Use of antidepressants and/or anticonvulsive agents	0 (0%)	0 (0%)		-
Scores for CNO-related bone pain pre-treatment (NRS 0–10)*				
Maximal pain in past 7 days, mean ± SD	5.4 ± 2.7	7.4 ± 1.6	1.9 (0.7–3.1)	0.005
Minimal pain in past 7 days, mean ± SD	2.2 ± 2.2	3.6 ± 2.1	1.3 (− 0.01–2.7)	0.064
Average pain in past 7 days, mean ± SD	4.0 ± 2.6	5.8 ± 1.6	1.8 (0.2–3.3)	0.008
Sleep disturbance in past 7 days, mean ± SD	2.2 ± 2.5	5.7 ± 2.7	3.5 (1.9–5.0)	< 0.001

*Level of significance set at 0.01 to adjust for multiple testing for pain score analyses

### Association of Mixed Pain with Patient-Reported Treatment Outcomes

**Fig. 4:**
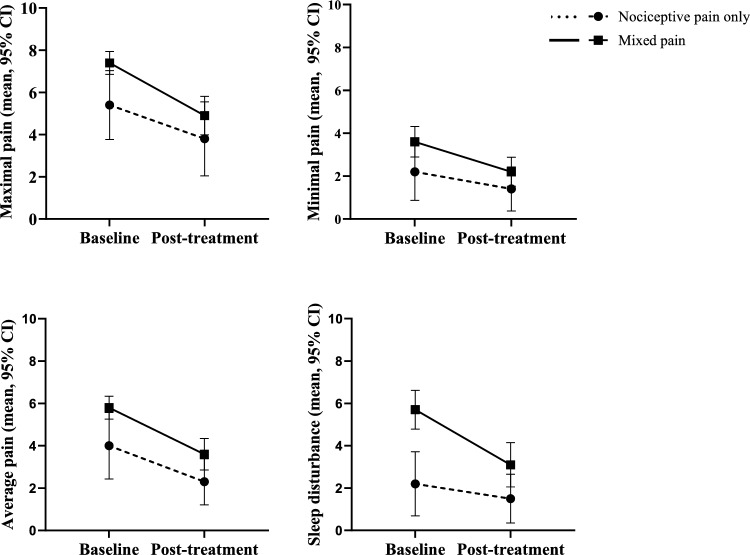
Changes in CNO-related bone pain scores after standard-of-care treatment with NSAIDs and/or bisphosphonates stratified for nociceptive pain only (*n* = 13) and mixed pain (i.e., nociceptive pain plus neuropathic pain and/or nociplastic pain) (*n* = 36)

CNO-related bone pain scores were compared before and after standard-of-care treatment with NSAIDs and/or bisphosphonates for patients with nociceptive pain only versus patients with mixes pain (Fig. 4). Analysis of covariance showed that the post-treatment score for maximal CNO-related bone pain adjusted for pre-treatment scores was nondifferential between groups (adjusted mean 4.1 and 4.6 for nociceptive and mixed pain, respectively, *p* = 0.634). Similarly, no differences between groups were found for minimal pain (adjusted mean 1.4 versus 2.1, *p* = 0.232), average pain (adjusted mean 2.6 versus 3.4, *p* = 0.269) or sleep disturbance (adjusted mean 2.6 versus 2.7, *p* = 0.922). However, post-treatment score for sleep disturbance remained significantly higher in the mixed pain group (mean difference 1.6, 95% CI 0.1–3.0, *p* = 0.037).

### Pain Management Strategies Outside Hospital Setting

**Fig. 5:**
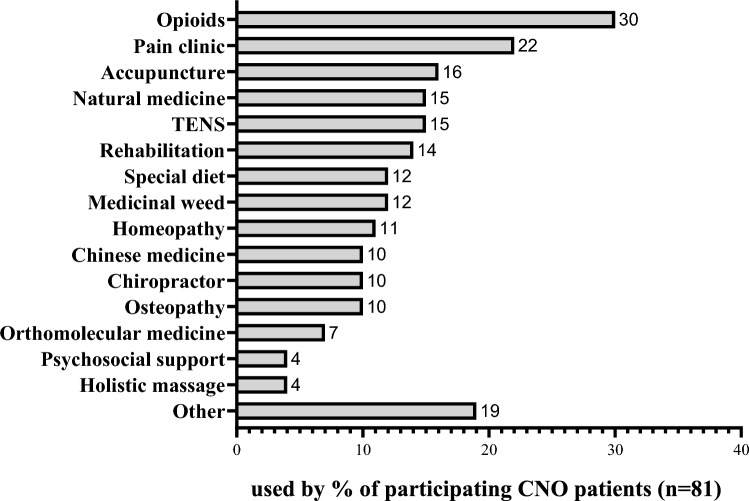
Use of pain management strategies by adult CNO patients outside the referral clinic (total *n* = 81)

Of the responding patients, *n* = 36, 44% indicated to seek/have sought additional pain treatment, either as additions to, or replacements for treatments received at the referral hospital. This group consisted mostly of patients with mixed pain (i.e., nociceptive plus neuropathic and/or nociplastic pain; 81%), and reported a variety of treatments (Fig. 5). *N* = 24, 30% of participants had been or were currently treated with opioids, for a median of 6 months (range 1–144 months). In 67% of patients opioids were prescribed by the general practitioner. Of opioid users, 71% received tramadol, 50% oxycodone (sustained and immediate release formulation), 4% transdermal fentanyl and 8% morphine. Among (ex)-opioid users, 18% fulfilled the criteria for a disorder in opioid use according to the diagnostic and statistical manual of psychiatric disorders (DSM)-5 criteria during their use [30]. Other common out-of-hospital pain management strategies included consultation at a pain clinic (*n* = 19, 22%), acupuncture (*n* = 13, 16%), natural medicine (*n* = 12, 15%), rehabilitation programmes (*n* = 11, 14%), Transcutaneous Electrical Nerve Stimulation (TENS) (*n* = 12, 15%) and medicinal weed (*n* = 10, 12%).

## Discussion

In this study, we found that the co-existence of neuropathic and nociplastic pain alongside nociceptive bone pain is common in adult CNO. Only 29% of participating patients had nociceptive pain exclusively. By contrast, the majority of patients reported mixed pain, which included combinations of nociceptive and neuropathic pain (3%), nociceptive and nociplastic pain (40%), or all three pain profiles (29%). These findings suggest that disease burden in CNO–largely dictated by pain–involves more than just nociceptive pain signals resulting from inflammation. Therefore, multi-angled rather than just anti-inflammatory treatments may offer opportunity to reduce the high burden of disease [6]. This notion is gaining recognition in other musculoskeletal diseases too [10, 17, 31–34], with growing understanding that nociceptive pain can intermingle with both neuropathic pain and nociplastic pain and integrated management can significantly improve patient outcomes [35].

Our study revealed that 32% of adult CNO patients exhibited neuropathic pain in addition to their nociceptive bone pain, as evaluated by PAIN-detect. This proportion is similar to what has been reported for axial spondylarthritis, which shares clinical overlap with CNO, where PAIN-detect scores range between 28% and 34% [18, 36, 37]. Although our data cannot reveal the exact source of neuropathic pain in CNO, it is known from other bone pathologies that neuropathic pain may arise due to damage to small nerve fiber in the bone and periosteum [38]. In CNO, this damage may arise from progressive sclerosis and hyperostosis, both of which characterize prolonged disease. Also, soft tissue ossification forms a key disease process and may cause small nerve fiber damage in the affected areas [39–41].

A majority of 69% of CNO patients in the present study displayed a nociplastic pain profile alongside their nociceptive pain, as reflected by signs of central sensitization on CSI and/or symptoms consistent with fibromyalgia on FiRST or the AAPT criteria, both of which represent elements of nociplastic pain [15]. These findings align with those from multiple studies on axial spondylarthritis and a single study on adult CNO [19, 42–45]. While nociplastic pain is described as pain that does not exhibit clear nociceptor activation or neuropathy [15], it is known to frequently develop within the context of chronic nociceptive pain via adaptive alterations in pain processing. Consequently, this commonly results in mixed pain including elements of both [15]. Inflammatory musculoskeletal diseases forms an illustrative example of this phenomenon, as inflammatory nociceptive pain often becomes entangled with nociplastic pain, sometimes taking the clinical shape of comorbid fibromyalgia [42]. Our data suggest that CNO is no exception to this phenomenon. Although this study does not enable us to draw causal conclusions about why CNO patients are prone to nociplastic pain, we can hypothesize about potential disease-specific risk factors. First, although this was not addressed in the study surveys, we speculate that the lack of awareness of the disease, and the absence of evidence-based treatments and clear prognostic information may be risk factors as they decrease patients’ perceived locus of control, which is known to play a role in nociplastic pain [46–48]. Second, diagnostic delays may be another risk factor for nociplastic pain development via both physiological and psychological mechanisms. Physiologically, persistent nociceptive signaling due to untreated disease activity is known to trigger the aforementioned plastic changes in the central nervous system, resulting in a widespread, intense pain phenotype [15, 35]. Psychologically, diagnostic delay is known to represent a period of distress, uncertainty and reduced self-efficacy, all of which are risk factors for the development of nociplastic pain [46–48]. Indeed, this study demonstrated that patients with mixed pain had longer diagnostic delays, confirming the suspected association. Although additional studies, preferably with in-depth patient interviews, are required to understand these mechanisms in more detail, it is once again evident that quick diagnosis of CNO should receive attention in both clinical practice and research.

Patients with mixed pain demonstrated higher CNO-related bone pain scores as compared to patients with exclusive nociceptive pain, even though disease severity as reflected by number of bone lesions, skeletal distribution pattern, and extra-skeletal features was similar between groups. This finding holds significance given that the current evaluation of CNO and therapeutic decision-making mainly relies on pain scores, owing to the absence of concrete biomarkers for measuring disease activity [2]. Our study therefore suggests that the presence of mixed pain complicate the interpretation of pain scores and their accuracy in reflecting the state of inflammation. Indeed, in other musculoskeletal diseases, mixed pain is increasingly recognized as an important contextual factor in the evaluation of disease activity [33, 49], associated with higher pain scores despite similar levels of inflammatory activity [50–52].

Patients with mixed pain exhibited similar or even greater improvements in CNO-related bone pain after treatment with NSAIDs and intravenous bisphosphonates, compared to those with only nociceptive pain. Initially, this result seemed surprising since these treatments are not typically considered effective against neuropathic nor nociplastic pain. However, the greater improvement in the mixed pain group can be attributed to their higher baseline pain levels, which inherently allow for greater decrease in psychometric sense. This pattern is also observed in other musculoskeletal conditions, where patients with mixed pain present higher pain scores at baseline, but achieve similar remission rates [53, 54]. Another explanation may be that patients with mixed pain more frequently receive intravenous bisphosphonates, which may be more effective against CNO-related bone pain than NSAIDs alone, and have also been suggested partly effective against neuropathic and/or nociplastic pain [1, 55].

A significant proportion of CNO patients, mainly those with mixed pain, had engaged in myriad pain-related therapies outside the hospital setting. Non-hospital pain treatments are common in other chronic musculoskeletal diseases as well, but they might hold a special appeal for CNO patients considering the absence of evidence-based treatments available within the clinical setting. While many of these interventions were unharmful, it is worth noting that opioids were used by 30% of patients at some point during disease course for a median duration of 6 months, and their usage was associated with the presence of mixed pain. The study design cannot ascertain whether opioid use precedes or follows the development of mixed pain, but underscores the importance of actively discussing these medications during patient evaluation. This is particularly crucial given the significant adverse effects and the risk of patient tolerance and misuse. Additionally, this finding calls for clinical collaboration with specialized pain centers which can provide expert guidance to patients on safely discontinuing opioid use and are specialized in pain medication effective for neuropathic and nociplastic pain.

There are several limitations to consider in this study. Firstly, CNO-related pain scores at baseline and after treatment were obtained from a different point in time compared to survey completion, which may have introduced variability in the data. In future studies, it is essential to implement systematic data collection from baseline and throughout follow-up, incorporating standardized measurements of pain scores, the extent of neuropathic and nociplastic pain, and narrow tracking of CNO and specific pain-related treatments. Such approaches offer improved insights into causal risk factors for mixed pain in adult CNO and consequently facilitate the implementation of preventive measures. Secondly, the sample size was small. However, it was reasonably substantial considering the rarity of the condition and the final sample effectively represented the CNO population in terms of sociodemographic and disease-specific characteristics. Thirdly, some relevant data were unavailable, such as health beliefs, religious background, and current socioeconomic status, all of which are recognized as factors associated with the development of mixed pain. Finally, although validated surveys were employed to classify pain types among patients, this approach has its constraints. Future studies should aim to integrate direct clinical data gathering, encompassing findings from physical examinations and neurophysiological pain evaluation methods like quantitative sensory testing.

Our findings have important implications for the clinic and research. Clinically, they imply that management for CNO needs to encompass more than just bone inflammation control. Measuring neuropathic and nociplastic pain via validated questionnaires, and addressing their presence in a multifaceted management plan may be an important step towards improving clinical outcomes, including work participation and overall quality of life [56–59]. Similarly, neuropathic and nociplastic pain should be considered as contextual factors in disease activity assessment and treatment decisions, as they may lead to overestimation of inflammatory activity and therefore over-treatment. Regarding research, we recommend to consider neuropathic pain and nociplastic pain in the design of treatment trials, which are anticipated in the near future to address the unmet need of evidence-based therapy in this rare disease.

### Supplementary Information

Below is the link to the electronic supplementary material.

Supplementary file1 (DOCX 26 KB)

Supplementary file2 (DOCX 13 KB)

## Data Availability

The datasets generated during and/or analyzed during the current study are available from the corresponding author on reasonable request.

## Abbreviations

AAPT: ACTION-APS pain taxonomy
ACW: Anterior chest wall
BPI: Brief pain inventory
CNO: Chronic nonbacterial osteitis
CSI: Central sensitization inventory
CsDMARDs: Conventional synthetic disease modifying anti-rheumatic drugs
FiRST: Fibromyalgia rapid screening tool
NRS: Numerical rating scale
NSAIDs: Non-steroidal anti-inflammatory drugs
PROM: Patient reported outcome measure
PPP: Pustulosis palmoplantaris
SAPHO: Synovitis, acne, pustulosis, hyperostosis, osteitis
