# Supplementary material for: Neuropathic and Nociplastic Pain Profiles are Common in Adult Chronic Nonbacterial Osteitis (CNO)
Source: Calcif Tissue Int. 2024 Apr 16;114(6):603–13. doi: 10.1007/s00223-024-01214-3 (PMC11090977; doi:10.1007/s00223-024-01214-3)
Supplement: Supplementary file 1 — Supplementary file1 (DOCX 26 KB) [file 223_2024_1214_MOESM1_ESM.docx]

S1: Questionnaire on Painkiller Use and Pain Treatments for CNO

Methodological note:

The questionnaire on painkiller use and pain treatments was simultaneously distributed with a questionnaire on the use of physical therapy in adult CNO, of which the data are reported elsewhere (1). The questionnaire originally sent in Dutch, and translated into English for publication purposes. Language level of B1 was established by the medical ethical review board of the Leiden University Medical Center.

**Introduction**

This questionnaire is about your use of painkillers or other pain treatments for the condition CNO. We would like to understand the various forms of pain treatment applied for CNO, including treatments that occur outside the hospital.

Please note: This questionnaire only pertains to painkiller use or pain treatment for CNO. Other conditions that cause pain (such as hernias, fractures, or abdominal pain) are not considered. Sometimes, there may be pain complaints alongside CNO that are closely related, such as joint inflammation (arthritis) or conditions like axial spondyloarthritis (SpA). If these symptoms are related to CNO, please complete the questionnaire for the entire condition.

Thank you for completing the questionnaire!

**Questionnaire**

The first part of the questionnaire is about the types of painkillers you have used or are currently using for CNO.

1. Have you used or are you currently using anti-inflammatory drugs for CNO? By this, we mean "NSAIDs," which include medications like ibuprofen, naproxen, diclofenac, indomethacin (Indocid), celecoxib (Celebrex), etoricoxib (Arcoxia), and meloxicam.

Yes / No / I don't remember

Did you use these anti-inflammatory drugs in a fixed dosage or varying amounts?
Fixed dosage / Varying amounts or as needed / Periods of fixed and varying

How long have you been using or did you use anti-inflammatory drugs for CNO?
___ months

Which anti-inflammatory drug did you use or are currently using?
Ibuprofen / Naproxen / Diclofenac / Indomethacin (Indocid) / Celecoxib (Celebrex) / Etoricoxib (Arcoxia) / Meloxicam

Did you use a stomach protector (such as omeprazole or pantoprazole) with your anti-inflammatory drugs?
Yes / No

Are you currently using anti-inflammatory drugs for CNO?
Yes / No

If not, what is the reason you stopped using anti-inflammatory drugs for CNO? You can select multiple reasons.
I no longer have CNO symptoms / Anti-inflammatory drugs did not help with CNO / I couldn't tolerate the anti-inflammatory drugs / I received a different treatment that made anti-inflammatory drugs unnecessary / My doctor advised discontinuation / I don't know / Other, please specify: (free text)

1. Have you used or are you currently using disease-modifying antirheumatic drugs (DMARDs) for CNO? This includes medications like methotrexate, sulfasalazine, plaquenil, and leflunomide.

Yes / No / I don't remember

How long have you been using or did you use DMARDs for CNO?

___ months

Which DMARD did you use or are currently using? You can select multiple options.
Methotrexate / Sulfasalazine / Plaquenil / Leflunomide

Are you currently using DMARDs for CNO?
Yes / No

If not, what is the reason you stopped using DMARDs for CNO? You can select multiple reasons.
I no longer have CNO symptoms / DMARDs did not help with CNO / I couldn't tolerate DMARDs / I received a different treatment that made DMARDs unnecessary / My doctor advised discontinuation / I don't know / Other, please specify:

1. Have you used or are you currently using bisphosphonates for CNO? This includes medications like pamidronic acid (APD), ibandronic acid, and zoledronic acid (Aclasta).

Yes / No / I don't remember

How long have you been using or did you use bisphosphonates for CNO?
___ months

Which bisphosphonate did you use or are currently using? You can select multiple options.
Pamidronic acid (APD) / Ibandronic acid / Zoledronic acid (Aclasta) / Other

Are you currently using bisphosphonates for CNO?
Yes / No

If not, what is the reason you stopped using bisphosphonates for CNO? You can select multiple reasons.
I no longer have CNO symptoms / Bisphosphonates did not help with CNO / Side effects were too severe / I received a different treatment that made bisphosphonates unnecessary / My doctor advised discontinuation / I didn't want to use bisphosphonates for too long / I don't know / Other, please specify:

1. Have you used or are you currently using biological drugs for CNO? This includes medications like infliximab (Remicade, Inflectra), golimumab (Simponi), adalimumab (Humira), and etanercept (Enbrel).
   Yes / No / I don't remember

How long have you been using or did you use biological drugs for CNO?
___ months

Which biological drug did you use or are currently using? You can select multiple options.
Infliximab (Remicade, Inflectra) / Golimumab

Are you currently using biologicals for CNO?
Yes / No

If not, what is the reason you stopped using biologicals for CNO? You can select multiple reasons.
I no longer have symptoms of CNO / Biologicals did not help with CNO / The side effects were too severe / I received a different treatment that made biologicals no longer necessary / My doctor advised stopping / I don't know / Other, please specify:

1. Have you used opioids for CNO or are you currently using them? This includes medications such as tramadol, oxycodone (Oxynorm, Oxycontin), fentanyl (patches), buprenorphine (patches), morphine.
   Yes / No / I don't remember

How long have you used opioids for CNO or are you currently using opioids for CNO?
___ months

Which opioids have you used or are using? You can select multiple options.
Tramadol / Oxycodone (Oxynorm, Oxycontin) / Fentanyl (patches) / Morphine / Other

Who prescribed the opioids?
General practitioner / Hospital doctor / Other

Are you currently using opioids for CNO?
Yes / No

If not currently using, what is the reason you stopped using opioids for CNO? You can select multiple reasons.
I no longer have symptoms of CNO / Opioids did not help with CNO / The side effects were too severe / I received a different treatment that made opioids no longer necessary / My doctor advised stopping / I don't know / Other, please specify...

Did you need help to stop using opioids?
No, I stopped on my own / Yes, I received guidance from my general practitioner / Yes, I received guidance from the hospital specialist / Yes, I received guidance from an addiction clinic / I would have liked help but couldn't find it anywhere.

If currently using, would you like to stop using opioids for CNO?
No / Yes

If yes, what do you see as possible obstacles to stopping opioids for CNO?
Free text

With strong painkillers like opioids, dependence can occur. Users may strongly desire the drug, and "tolerance" can develop, meaning higher doses of opioids are needed to achieve the same pain relief. We would like to know if you have experienced or are experiencing these effects with opioid use. We understand that these are difficult and uncomfortable questions to answer. If you prefer not to answer, you can leave the question blank and proceed to question 7.

I often use(d) opioids in higher doses or for longer than intended. Yes / No

I had the desire to reduce or control opioid use, or I made unsuccessful attempts to do so. Yes / No

I spent/spend a lot of time obtaining opioids, using opioids, or recovering from their effects. Yes / No

I experience(d) cravings, a strong desire, or urge to use opioids. Yes / No

Due to opioid use, I could/can't fulfill important obligations at work, school, or at home. Yes / No

Opioid use persisted/persists despite social or personal problems it caused. Yes / No

Important social, work, or leisure activities were stopped or reduced due to opioid use. Yes / No

Opioid use sometimes posed poses a physical danger. Yes / No

I continue(d) to use opioids even though I know there is a persistent or recurring physical or psychological problem likely caused or worsened by opioids. Yes / No

I needed a higher dose/growing amount of opioids to achieve the desired effect, or I experienced a clear diminished effect when using the same dose for a while. Yes / No

I experience(d) withdrawal symptoms when I don't use opioids for a while (e.g., agitated mood, nausea, vomiting, pain, sweating, diarrhea, yawning), or I have to use opioids at such times to reduce these symptoms. Yes / No

1. Have you ever undergone a rehabilitation program for CNO?

Yes / No / I don't remember

How long did this program last?
___ months

Where did you undergo the program?
At a nearby rehabilitation center / At a rehabilitation department of the hospital / Other

What was the effect of the rehabilitation program for CNO on the following areas?
0 = significant worsening of symptoms 1 = a slight worsening of symptoms 2 = no effect on symptoms 3 = a slight improvement in symptoms 4 = significant improvement in symptoms.
Pain: 0-4
Function (use of the body during activities or tasks at home): 0-4
Physical activity: 0-4
Sleep: 0-4
Quality of life: 0-4
Psychological well-being: 0-4

1. Have you ever been referred to a pain clinic for CNO, possibly at your own request?
   Yes, by my general practitioner / Yes, by my hospital doctor / No
2. Have you ever been treated with a Transcutaneous Electrical Nerve Stimulation (TENS) device for CNO?
   Yes/no

If yes, how long have you used or are you currently using the TENS for CNO?

___ months

Are you currently using the TENS for CNO?
Yes / No

If no, what is the reason you stopped using the TENS for CNO? You can select multiple reasons.
I no longer have symptoms of CNO / The TENS did not help with CNO / I could not tolerate the TENS / I received a different treatment that made the TENS no longer necessary / My doctor advised stopping / I don't know / Other, please specify.

1. Have you ever tried the following treatments for your CNO symptoms? You can select multiple options:

Homeopathy

Chinese medicine

Naturopathy (if yes, specify in free text)

Acupuncture

Ayurveda

Anthroposophic medicine

Chiropractic

Haptotherapy

Orthomolecular medicine

Osteopathy

Psychosocial therapy

Holistic massage

CBD oil / medical cannabis

Modified diet

Other, please specify

None of the above

The following questions are about pain. This includes bone pain from CNO, joint pain from possible arthritis, muscle and ligament pain, or pain of unknown origin. You can exclude pain that you clearly know is caused by something temporary (such as an eye infection, menstrual pain, a sore throat, or a broken arm).

1. In which locations do you experience pain? You can select multiple options.

Region of the sternum, upper ribs, and collarbones / chest

Jaw

Head

Left arm

Right arm

Abdomen

Upper back and spine

Lower back and spine and buttocks

Right leg

Left leg

1. Indicate whether the following statements apply to you:

I experience moderate to severe sleep problems OR fatigue due to my pain symptoms (yes/no)

These pain symptoms have been present for more than 3 months (yes/no)

1. Indicate whether the following statements apply to you (yes/no)

I have pain throughout my entire body

My pain is accompanied by continuous and unpleasant overall fatigue

My pain feels like burning, electric shocks, or cramps

My pain is accompanied by other unusual sensations in my body, such as tingling and numbness

My pain is accompanied by other health problems such as gastrointestinal issues, urinary problems, headaches, or restless legs

My pain has a significant impact on my life, especially on my sleep and concentration, and makes me feel sluggish

1. Leerling AT UD, Van der Giesen FJ , Vliet Vlieland TPM, Winter EM. The role of physical therapy in adult chronic nonbacterial osteitis (CNO): patients’ and therapists’ perspective. Under revision.
