# Supplementary material for: Neuropathic and Nociplastic Pain Profiles are Common in Adult Chronic Nonbacterial Osteitis (CNO)
Source: Calcif Tissue Int. 2024 Apr 16;114(6):603–13. doi: 10.1007/s00223-024-01214-3 (PMC11090977; doi:10.1007/s00223-024-01214-3)
Supplement: Supplementary file 2 — Supplementary file2 (DOCX 13 KB) [file 223_2024_1214_MOESM2_ESM.docx]

**Table :** Correlation of scores for neuropathic pain on PAIN-detect and nociplastic pain on CSI and scores for CNO-related bone pain. *** p<0.0125, for Pearson’s *r* (adjusted level of significance) . CSI = central sensitization inventory.

| **Scores for CNO-related bone pain (NRS 0-10)** | **Neuropathic pain as scored by PAIN-Detect (0-38)** | **Nociplastic pain as scored by CSI (0-100)** |
| --- | --- | --- |
| Maximal pain in past 7 days | 0.383*** | 0.359*** |
| Minimal pain in past 7 days | 0.394*** | 0.204 |
| Average pain in past 7 days | 0.413*** | 0.314 |
| Sleep disturbance in past 7 days | 0.566*** | 0.457*** |
